# SARS-CoV-2 vaccinations reduce the prevalence of post-COVID Guillain-Barre syndrome

**DOI:** 10.1016/j.clinsp.2022.100064

**Published:** 2022-06-13

**Authors:** Josef Finsterer, Daniel Matovu, Fulvio A. Scorza

**Affiliations:** a Neurology & Neurophysiology Center, Vienna, Austria; b Disciplina de Neurociência, Universidade Federal de São Paulo/Escola Paulista de Medicina (UNIFESP/EPM), São Paulo, SP, Brasil

**Keywords:** SARS-CoV-2, COVID-19, Neuro-COVID, Complications, Polyradiculitis, Vaccination, AIDP, Acute, Inflammatory, Demyelinating Polyneuropathy, AMAN, Acute, Motor Axonal Neuropathy, AMSAN, Acute, Motor and Sensory Axonal Neuropathy, BSE, Bickerstaff encephalitis, CSF, Cerebro-spinal fluid, GBS, Guikkaub Barre syndrome, IVIG, Intravenous Immunoglobulins, PCB, Pharyngeal, Cervical, and Brachial variant, PCG, Post COVID-19 GBS, PNC/MNC, Poly- or Mono-Neuritis Cranialis

## Abstract

Guillain-Barre Syndrome (GBS) has been repeatedly reported as a neurological complication of COVID-19 (post-COVID GBS [PCG]). Whether the introduction of SARS-CoV-2 vaccines reduced the prevalence of PCG is unknown. This narrative review aimed to compare the number of published PCG cases between the second half of 2020 (no vaccination available) with those of the first half of 2021 (vaccination available). A total of 124 articles reported 300 patients with PCG between January 2020 and June 2021. The ages ranged from 7 to 94y. There was male dominance. The latency between the onset of COVID-19 and the onset of PCG ranged from -10 to 90d Acute, inflammatory, demyelinating polyneuropathy was diagnosed in 171 patients, acute, motor axonal neuropathy in 24, and acute, motor, and sensory axonal neuropathy in 16 patients. Regarding treatment, 241 patients received immunoglobulins, 28 patients’ plasmaphereses, and 7 patients' steroids. Artificial ventilation was required in 59 patients. Full recovery was achieved in 42 cases, partial recovery in 163 cases, and 17 patients died. The number of published PCG patients fell from 192 in the second half of 2020 to 75 patients in the first half of 2021. It is concluded that the prevalence of PCG has decreased since the introduction of SARS-CoV-2 vaccines. SARS-CoV-2 vaccinations have a positive effect on the prevalence of PCG.

## Highlights

• SARS-CoV-2 infections can be complicated by Guillain-Barre Syndrome (GBS).
• The prevalence of SARS-CoV-2 associated GBS declined since the introduction of SARS-CoV-2 vaccines.
• The outcome of SARS-CoV-2 associated GBS is worse among those with comorbidities compared to those without.

## Introduction

Guillain-Barré Syndrome (GBS) is an increasingly perceived complication of SARS-CoV-2 (COVID-19) infections.^1^ In the first half of 2020, only a few patients with SARS-CoV-2 associated GBS (post-COVID GBS (PCG)) were published.^1^, ^2^, ^3^, ^4^, ^5^, ^6^, ^7^, ^8^, ^9^ In the second half of 2020 the number of published PCG patients increased significantly. Since December 2020 several brands of SARS-CoV-2 vaccinations have been launched. It is unknown whether the frequency of PCG has decreased since the introduction of these anti-SARS-CoV-2 vaccinations. Therefore, the present narrative, up-to-date review aimed to compare the number, demographics, clinical presentation, therapeutic management, and outcome of PCG in the 6 months before and after vaccine availability (July to December 2020 compared with January 2021 to June 2021) and to answer the question of whether SARS-CoV-2 vaccinations reduce the prevalence of PCG.

## Methods

A literature search in the databases PubMed and Google Scholar using the search terms “neuropathy”, “Guillain Barre syndrome”, “polyradiculitis”, “AIDP”, “AMAN”, “AMSAN”, “Miller-Fisher syndrome”, “polyneuritis cranialis”, “cranial nerve”, and “Bickerstaff encephalitis”, in combination with “SARS-CoV-2″, “COVID-19″, and “coronavirus” was conducted. Additionally, reference lists were checked for further articles meeting the search criteria. Included were only original articles detailing individual patients’ data (age, sex, latency between onset of COVID-19 and onset of GBS, GBS subtype, results of CSF investigations, comorbidities, treatment, and outcome) published between January 2020 and June 2021.^1^ Excluded from data analysis were reviews, abstracts, proceedings, and editorials. Cohort studies that did not provide sufficient individual data were also excluded.

## Results

**Fig. 1:**
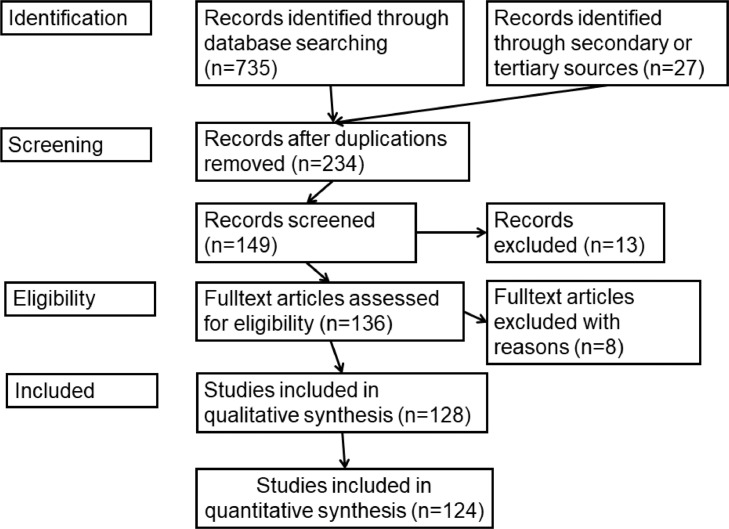
flowchart of the selection process upon which papers were included or excluded.

By the end of June 2021, a total of 124 articles were found that met the inclusion criteria and described individual patients with PCG (Fig. 1). The first patient with PCG was reported by Zhao et al. in May 2020.^10^^,^^11^ By the end of June 2020, 33 patients with PCG were published (Table 1). From July to December 2020, 192 PCG patients were published (Table 2). From January 2021 to the end of June 2021, a further 75 PCG patients were published (Table 3). The 124 articles published from early January 2020 to late June 2021 reported 300 patients with PCG (Table 4). Relevant data on age, gender, onset before/after COVID-19, latency between COVID-19 and onset of PCG, the subtype of GBS, PCR result in the Cerebrospinal Fluid (CSF), therapy, and outcome are presented in Table 4. The ages of these patients, available from 295 patients, ranged from 7 to 94y. The sex was male in 201 cases and female in 92 cases. The onset of PCG, available from 243 cases, was identified in 233/3/7 patients after/along with/before the onset of COVID-19. The latency between the onset of COVID-19 and the onset of PCG ranged from −10 and 90 days. The GBS subtype, reported in 233 cases, was identified as Acute, Inflammatory, Demyelinating Polyneuropathy (AIDP) in 171 patients, as Acute, Motor Axonal Neuropathy (AMAN) in 24, as Acute, Motor and Sensory Axonal Neuropathy (AMSAN) in 16, as Miller-Fisher Syndrome (MFS) in 8 patients, as Poly- or Mono-Neuritis Cranialis (PNC/MNC) in 3, and as Pharyngeal, Cervical, and Brachial (PCB) variant in 1 patient. Bickerstaff Encephalitis (BFE) was not reported in any case. SARS-CoV-2 was only detected in the CSF of a single patient. Therapy of PCG, available in 270 cases, included Intravenous Immunoglobulins (IVIG) in 241 patients, plasmapheresis in 28, steroids in 7, and no therapy in 8 cases. Artificial ventilation was required for 59 patients. The outcome, available from 222 cases, was rated as full recovery (*n* = 42), a partial recovery (*n* = 163), or death (*n* = 17). Comparing patients with and without comorbidities, the incidence of a fatal outcome was higher in those with comorbidities than in those without. Among those with comorbidities, 8 died and among those, without comorbidities, only 5 died. The comparison of the patients published in the second half of 2020 with the patients published in the first half of 2021 showed that the number of publications and thus the number of patients had fallen from 192 to 75 patients in the first half of 2021 (Table 4).

**Table 1:** Patients with PCG as reported by the end of June 2020.

**Age (y)**	**Sex**	**Onset**	**LOO (d)**	**Subtype**	**CIC**	**CM**	**Therapy**	**AV**	**Outcome**	**Country**	**Reference**
54	m	A	8	AIDP	nr	No	IG	Yes	Complete	USA	Virani 4/20
71	m	A	4	AIDP	No	AHT, AAR, LC	IG	Yes	Death	Italy	Alberti 4/20
46	m	A	18	AIDP	nr	nr	No	No	Partial	Iran	Ebrahimzadeh 4/21
65	m	A	10	AIDP	nr	nr	IG	no	Partial	Iran	Ebrahimzadeh 4/21
61	f	B	9	AIDP	nr	No	IG	No	Complete	China	Zhao 5/20
61	m	A	10	MFS	No	No	S	No	Complete	Spain	Juliao Caamano 5/20
76	f	A	8	GBS*	nd	No	No	nr	Death	Spain	Marta-Enguita 5/20
43	m	A	21	AIDP	No	nr	IG	No	Complete	France	Bigaut 5/20
71	f	A	10	AIDP	No	nr	IG	No	Partial	France	Bigaut 5/20
55	f	A	14	AIDP	nr	No	IG	Yes	Partial	Spain	Esteban Molina 5/20
61	f	A	7	AMAN	No	nr	IG	No	Partial	USA	Valiuddin 5/20
43	m	A	10	AIDP	nr	nr	IG	No	Partial	Spain	Velayos Galan 5/20
58	m	AB	0	AIDP	No	No	IG	No	Partial	Canada	Chan 5/20
68	m	A	13	nr	nr	nr	PE	No	Partial	USA	Chan 5/20
84	m	A	23	nr	nr	nr	PE, IG	Yes	Partial	USA	Chan 5/20
65	m	A	9	AMSAN	nd	DM	IG	No	nr	Iran	Sedaghat 6/20
66	f	A	7	AIDP	No	nr	IG	Yes	Complete	Italy	Ottaviani 6/20
54	f	A	21	AIDP	nd	No	IG	No	Complete	Germany	Scheidl 6/20
70	f	A	3	AMSAN	No	RA	IG	No	Partial	Morocco	El Otmani 6/20
64	m	A	11	AIDP	nd	No	IG	Yes	nr	France	Camdessanche 6/20
nr	nr	A	7	AIDP	No	nr	IG	No	Partial	Italy	Toscano 6/20
nr	nr	A	10	AIDP	No	nr	IG	No	Complete	Italy	Toscano 6/20
nr	nr	A	10	AMAN	No	nr	IG	Yes	Partial	Italy	Toscano 6/20
nr	nr	A	5	AMAN	No	nr	IG	No	Partial	Italy	Toscano 6/20
nr	nr	A	7	AMAN	No	nr	IG, PE	No	nr	Italy	Toscano 6/20
54	m	A	14	AIDP	Nd	nr	IG	No	Partial	USA	Rana 6/20
53	f	B	nr	AIDP	No	No	PE	No	Partial	Turkey	Oguz-Akarso 6/20
51	f	A	14	MFS	nr	nr	IG	No	Partial	Spain	Reyes-Bueno 6/20
68	m	A	14	AIDP	nr&	nr	IG, PE	Yes	Partial	Austria	Helbok 6/20
53	m	A	24	AIDP	No	No	IG	No	Complete	Netherlands	Kilinc 6/20
57	m	A	6	AIDP	No	AHT, psoriasis	IG	Yes	Partial	UK	Webb 6/20
21	m	A	16	AIDP	nr	AHT, DM	PE	No	Complete	USA	Hutchins 6/20
41	m	A	10	AIDP	nr	DM	IG	No	Partial	Iran	Farzi 6/20

A, Onset of GBS after onset of non-neurological manifestations, AAR, Aortic Aneurysm Repair, AHT, Arterial Hypertension; AL, Alcoholism; AV, Artificial Ventilation; B, Onset of GBS before onset of non-neurological manifestations; CA, Carcinoma; CHD, Coronary Heart Disease; CIC, CoV2 in CSF; CM, Comorbidities, COPD, Chronic Obstructive Pulmonary Disease; DM, Diabetes; f, female; GBS*, no NCSs reported; HLP, Hyperlpidemia; IG, Immunoglobulins; LC, Lung Cancer; LOO, Latency between onset of GBS and COVID-19 respectively vice versa; m, male; nd, not done; nr, not reported, NTX, Renal Transplantation; pc, personal communication; PCB, Pharyngeal, Cervical, Brachial variant of GBS; PE, Plasma Exchange; PNC, Polyneuritis Cranialis; RA, Rheumatoid Arthritis; RI, Renal Insufficiency; RSD, Reflex Sympathetic Dystrophy; S, Steroids, & antibodies positive in CSF.

**Table 2:** Patients with PCG as reported from July to December 2020.

**Age (y)**	**Sex**	**Onset**	**LOO (d)**	**Subtype**	**CIC**	**CM**	**Therapy**	**AV**	**Outcome**	**Country**	**Reference**
56	f	A	15	AIDP	No	Nr	IG	Yes	Partial	Spain	Sancho-Saldana 7/20
70	f	A	23	AIDP	nd	No	IG	yes	Nr	Italy	Padroni 7/20
∼75	m	B	10	AIDP	No	No	IG	no	Complete	Swiss	Coen 7/20
64	m	A	23	AIDP	No	Nr	IG	No	Complete	France	Arnaud 7/20
36	m	A	4	MFS	nr	Nr	IG	No	Complete	USA	Lantos 7/20
55	m	A	20	AIDP	No	Nr	IG	Yes	Partial	Italy	Assini 7/20
60	m	A	3	AMSAN	No	Nr	IG	Yes	Partial	Italy	Assini 7/20
38	m	A	16	AIDP	nr	AHT	PE	No	Complete	Iran	Paybast 7/20
14	f	A	nr	nr	nr	No	IG	No	Complete	Iran	Paybast 7/20
49	m	A	14	AIDP	No	No	IG	No	Complete	UK	Tiet 7/20
68	m	A	5	AIDP	nr	AHT, HLP	IG	No	Complete	Italy	Agosti 7/20
11	m	A	21	AIDP	nr	No	IG	No	Complete	Saudi	[Khalifa 7/20]
15	m	A	nr	AMAN	No	No	IG	No	Partial	Brazil	Frank 7/20
72	m	A	18	AIDP	No	Nr	IG	Yes	Partial	Italy	Manganotti 7/20
72	m	A	30	AIDP	No	Nr	IG	yes	Partial	Italy	Manganotti 7/20
49	f	A	14	AIDP	No	Nr	IG	No	Partial	Italy	Manganotti 7/20
94	m	A	33	AIDP	nr	Nr	S	No	Partial	Italy	Manganotti 7/20
76	m	A	22	AIDP	No	Nr	IG	Yes	Partial	Italy	Manganotti 7/20
64	m	A	nr	nr	nr	DM	IG	Yes	Complete	Japan	Wada 7/20
77	m	A	nr	AIDP	nr	AHT, HLP	IG	no	Complete	Spain	Garcia-Manzanedo 7/20
75	m	A	nr	nr	No	Spinal trauma	IG	no	Complete	USA	Elkhouly 7/20
37	nr	A	10	nr	nr	Nr	nr	nr	nr	Belgium	Guilmot 7/20
51	m	A	12	AIDP	No	Nr	IG	Yes	Partial	Germany	Pfefferkorn 7/20
65	m	A	3	AIDP	nr	No	IG	No	Complete	Germany	Lampe 7/20
12	m	A	7	nr	nr	No	IG	Yes	Death	Tanzania	Manji 7/20
66	f	A	30	AIDP	nr	DM, AHT, arthritis	IG	No	Partial	Iran	Mozhadehipanah 7/20
55	f	A	31	AMSAN	nr	COPD	IG	Yes	Death	Iran	Mozhadehipanah 7/20
70	m	A	15	AMAN	nr	Nr	IG	No	Partial	Spain	Guijarro-Castro 7/20
34	m	A	4	PNC	nr	Strabism	IG	No	Partial	USA	Dinkin 8/20
71	f	A	Days	PNC	nr	AHT	no	No	Partial	USA	Dinkin 8/20
58	f	A	6	AIDP	No	Nr	PE	No	Complete	USA	Naddaf 8/20
56	f	A	7	AIDP	No	AHT, thyroxin ↓	nr	nr	Partial	Germany	Pelea 8/20
61	f	A	7	AMAN	No	AHT, DM, HLP, CA	S, PE	No	Complete	USA	Maideniuc 8/20
50	m	A	3	MFS, PNC	No	No	IG	No	Complete	Spain	Guttierez-Ortiz 8/20
39	m	A	3	MFS, PNC	No	No	No	No	Complete	Spain	Guttierez-Ortiz 8/20
72	m	A	7	AIDP	No	AHT, CHD, AL	IG	Yes	Partial	USA	Su 8/20
41	m	A	10	AIDP	No	Nr	IG	No	Complete	Guinea	Atakla 8/20
70	f	A	90	nr	nr	RSD	IG	No	Complete	USA	Defabio 8/20
57	m	A	17	AIDP	nr	Nr	IG	no	Partial	Italy	Zito 8/20
63	m	A	1	MFS	nr	Nr	no	No	Partial	UK	Ray 8/20
65	m	A	5	AIDP	nr	DM, AHT	IG	Yes	Death	Sudan	Sidig 8/20
62	m	A	12	nr	nr	COPD, sleep apnea	IG	Yes	Partial	UK	Jones 8/20
∼65	m	A	17	AIDP	No	No	IG	No	Complete	Italy	Riva 9/20
52	f	A	15	AIDP	No	Nr	IG	No	Partial	Swiss	Lascano 9/20
63	f	A	7	AIDP	nr	nr	IG	No	Complete	Swiss	Lascano 9/20
61	f	A	22	AIDP	No	nr	IG	No	Partial	Swiss	Lascano 9/20
74	f	A	nr	AIDP	No	Lymphoma	IG	No	Complete	Spain	Fernandez-Doming 9/20
∅53	11m	A	0.5‒28	AIDP	No, *n* = 4	nr	IG, *n* = 15	nr	Partial,	Italy, *n* = 17	Foresti 9/20
							PE, *n* = 2		Death, *n* = 1		
58	f	A	14	nr	nr	disk prolapse	IG	No	Partial	USA	Korem 9/20
∼35	m	A	nr	AMAN	No	nr	IG	No	Partial	UK	Ameer 9/20
49	m	A	11	PCB	No	AHT, seminoma	no	No	Partial	Italy	Liberatore 9/20
70	f	A	15	AMAN	nr	AHT, obesity	IG, PE	No	Partial	Italy	Masuccio 9/20
48	m	A	18	AIDP	nr	DM	PE	No	Partial	USA	Granger 9/20
55	f	A	10	AMAN	nr	CM, AHT	IG	No	Partial	India	Nanda 9/20
72	m	A	4	AIDP	nr	AHT	IG	Yes	Death	India	Nanda 9/20
55	m	A	4	AMSAN	nr	AHT, DM, dialysis	IG	No	Partial	India	Nanda 9/20
49	m	A	6	AIDP	nr	AHT	IG	No	Partial	India	Nanda 9/20
67	f	A	10	nr	No	Breast cancer	PE	Yes	Partial	USA	Abrams 10/20
76	m	A	7	AIDP	No	Cardiomyopathy	IG	No	Partial	France	Tard 10/20
44	m	A	nr	nr	nr	AHT, asthma	IG	No	Complete	USA	Khaja 10/20
54	f	A	20	AMAN	nr	Asthma	No	No	Partial	Japan	Hirayama 10/20
55	f	A	11	AMSAN	nr	Lung disease	IG	Yes	Death	Iran	Agha Abbaslou 10/20
8	m	B	nr	AIDP	No	No	IG	Yes	Partial	USA	Curtis 10/20
54	f	AB	0	nr	No	AHT	IG	No	Partial	Spain	Redondo-Urda 10/20
54	m	A	4	AIDP	nr	AHT, obesity	IG	Yes	Partial	Spain	Diaz-Porras 10/20
20‒63	7m	nr	nr	AIDP	nr	nr	IG	No	Partial	UK, *n* = 7	Paterson 10/20
59	m	A	20	nr	nr	Renal transplant	IG	Yes	Partial	Pakistan	Yaqoob 10/20
49	m	A	14	MFS	No	Crohn's disease	IG	Yes	Partial	USA	Lowery 11/20
65	f	A	nr	AIDP	nr	Fibromyalgia	IG	Yes	Death	Italy	Ferraris 11/20
88	f	A	2	AMSAN	nr	nr	PE	Yes	Partial	Iran	Abolmaali 11/20
47	m	A	7	AMSAN	nr	nr	PE	Yes	Death	Iran	Abolmaali 11/20
58	m	A	9	AMSAN	nr	nr	IG, PE	Yes	Death	Iran	Abolmaali 11/20
54	m	A	3	nr	nr	GBS, DM	IG	No	Complete	USA	McDonnell 11/20
37	m	A	14	AIDP	nr	nr	IG	Yes	Partial	Iran	Boostani 11/20
65	m	A	nr	AIDP	nr	nr	IG	No	Partial	Italy	Garnero 11/20
73	m	AB	0	AIDP	No	nr	IG	No	Partial	Italy	Garnero 11/20
55	m	A	20	AIDP/ MFS	No	nr	IG	No	Partial	Italy	Garnero 11/20
46	f	A	3	AIDP	No	nr	IG	No	Partial	Italy	Garnero 11/20
60	m	A	20	AMSAN	No	nr	IG	No	Partial	Italy	Garnero 11/20
63	f	A	15	AMSAN	nr	nr	IG	No	Partial	Italy	Garnero 11/20
66	f	B	No symptom	AIDP	nr	nr	IG	No	Partial	Italy	Bracaglia 11/20
54	nr	nr	nr	nr	No	AHT, HLP	IG	Yes	Complete	Spain	Barrachina-Esteve 11/20
61	m	A	nr	MFS	No	nr	IG	No	Complete	Germany	Senel 11/20
58	m	B	nr	AIDP	nr	nr	IG	Yes	Partial	UK	Gale 11/20
∅ 59.2	22m	A	16‒35	AIDP, *n* = 23	nr	nr	IG, *n* = 25	*n* = 5	Partial	UK, *n* = 30	Filosto 11/20
				AMAN, *n* = 2			PE, *n* = 2	*n* = 25			
65	m	A	5	AIDP	nr	nr	IG	No	nr	India	Kushwaha 11/20
60	f	A	22	nr	nr	Migraine	IG	No	Partial	USA	Bueso 12/20
∅57	33m	nr	0‒37	nr	nr	nr	IG, *n* = 46	nr	Death, *n* = 1	UK, *n* = 47	Keddie 12/20
							PE, *n* = 1		nr, *n* = 46		
57	m	A	nr	AMAN	nr	nr	IG	No	nr	Italy	Petrelli 12/20
53	m	A	nr	nr	nr	nr	IG	No	Partial	Italy	Gigli 12/20
36	m	A	18	AIDP	nr	AHT, NTX	IG	Yes	Partial	USA	Rajdev 12/20
72	f	A	8	AIDP	No	nr	IG	Yes	Partial	Italy	Civardi 12/20
20	m	A	5	AMAN	nd	No	IG	No	Complete	India	Ghosh 12/20
64	m	A	21	VII palsy	nr	nr	no	No	Partial	USA	Judge 12/20
75	m	A	nr	AIDP	nr	nr	IG	Yes	Partial	India	Chakraborty 12/20

A, Onset of GBS after onset of non-neurological manifestations; AAR, Aortic Aneurysm Repair; AHT, arterial hypertension; AL, Alcoholism; AV, Artificial Ventilation; B, Onset of GBS before onset of non-neurological manifestations; CA, Carcinoma; CHD, Coronary Heart Disease; CIC, CoV2 in CSF; CM, Comorbidities; COPD, Chronic Obstructive Pulmonary Disease; DM, Diabetes; f, female; GBS*, No NCSs reported; HLP, Hyperlpidemia; IG, Immunoglobulins; LC, Lung Cancer; LOO, Latency between Onset of GBS and COVID-19 respectively vice versa; m, male; nd, not done; nr, not reported, NTX, Renal Transplantation; pc, personal communication; PCB, Pharyngeal, Cervical, Brachial variant of GBS; PE, Plasma Exchange; PNC, Polyneuritis Cranialis; RA, Rheumatoid Arthritis; RI, Renal Insufficiency; RSD, Reflex Sympathetic Dystrophy; S, Steroids, & antibodies positive in CSF.

**Table 3:** Patients with PCG reported from 1.1.2021 to 30.6.2021.

**Age (y)**	**Sex**	**Onset**	**LOO (d)**	**Subtype**	**CIC**	**CM**	**Therapy**	**AV**	**Outcome**	**Country**	**Reference**
72	m	A	12	nr	nr	No	IG	No	Partial	Morocco	Mansour 1/21
36	f	A	42	AIDP	no	Pregnant	IG	No	Complete	Morocco	Aasfara 1/21
62	m	A	20	AIDP	nr	AHT, obesity	IG	Yes	Partial	Italy	Colonna 1/21
46	m	A	53	AIDP	nr	No	IG	No	Partial	UK	Raahimi 1/21
55	f	A	nr	nr	nr	AHT	IG, S	nr	Death	India	Goel 2/21
17	m	A	nr	nr	nr	No	IG, S	nr	Death	India	Goel 2/21
35	m	A	16	nr	nr	nr	IG	No	Complete	USA	Yakoby 3/21
36	f	A	18	nr	nr	Obesity	IG	No	Partial	USA	Dufour 3/21
39	f	A	14	AIDP	nr	DM, AHT	PE	No	Partial	Colombia	Mackenzie 3/21
53	m	A	9	AMSAN	nr	DM	PE, IG	Yes	Partial	USA	Brown 3/21
45	m	A	nr	AMAN	nr	Hypothyroid	IG	No	Partial	nr	Singh 3/21
53‒65 (15)	13m	A	∅12	AIDP	nr	nr	IG	Yes (2)	Partial (14)	France, *n* = 15	Meppiel 3/21
61	m	A	21	AIDP	No	nr	IG	Yes	Partial	Italy	Avenali 3/21
72	f	A	8	AIDP	No	nr	IG, PE	No	Partial	Italy	Avenali 3/21
57	m	A	12	AMSAN	No	nr	IG	No	Partial	Italy	Avenali 3/21
35‒81	17m	A	∅ 28.5	AIDP	nr	AHT, DM, *n* = 3	nr	nr	nr	Italy, *n* = 24	Uncini 3/21
8	m	A	28	AMAN	No	no	IG	No	Partial	Chile	Sandoval 3/21
71	f	A	8	AIDP	nr	AHT, hypothyroid	IG	Yes	Partial	Belgium	Paradis 4/21
52	f	A	nr	AIDP	nr	nr	IG	Yes	Partial	Swiss	Epiney 4/21
70	m	A	nr	AMAN	nr	Sleep apnea	nr	Yes	Partial	Swiss	Epiney 4/21
34	f	A	9	AMSAN	No	No	IG	No	Partial	Turkey	Tekin 4/21
70	m	nr	nr	nr	nr	nr	IG	Yes	Death	Iran	Nejad 5/21
22	f	A	7	AIDP	nr	Pregnant	IG	No	Partial	Philippines	Garcia 5/21
7	m	B	nr	AMAN	nr	No	IG	Yes	Partial	India	Das 5/21
27	m	A	5	AMAN	No	nr	IG	No	Complete	India	Khan 6/21
35	f	A	9	AIDP	No	No	supportive	No	Complete	India	Khan 6/21
40	f	A	20	AIDP	No	nr	IG	Yes	Death	India	Khan 6/21
48	f	A	1	AIDP	No	Pericarditis	IG	No	Complete	India	Khan 6/21
50	m	A	2	AMSAN	Yes	nr	IG	No	Complete	India	Khan 6/21
29	f	A	9	AIDP	nr	Pregnant	IG	No	Complete	Iran	Mehrpour 6/21
62	m	nr	nr	AMSAN	No	nr	IG, PE	No	Partial	Italy	d'Orsi 6/21
70	m	A	15	AMAN	nr	COPD, CHD,	IG, PE	Yes	Death	Turkey	Koca 6/21
34	m	A	10	AIDP	nr	nr	PE, IG	Yes	Partial	Egypt	Khedr 6/21
65	m	A	5	AIDP	nr	Cerebellar bleeding	PE	No	Partial	Egypt	Khedr 6/21
49	f	A	3	AMAN	nr	nr	PE, IG	No	Partial	Egypt	Khedr 6/21
45	m	A	14	AIDP	nr	nr	S	No	Partial	Egypt	Khedr 6/21
55	f	A	14	AMAN	nr	nr	IG	No	Partial	Egypt	Khedr 6/21
11	f	A	nr	AMAN	nr	No	IG, S, PE	Yes	Partial	India	Khera 6/21

A, Onset of GBS after onset of non-neurological manifestations; AAR, Aortic Aneurysm Repair; AHT, Arterial Hypertension; AL, Alcoholism; AV, Artificial Ventilation; B, Onset of GBS before onset of non-neurological manifestations; CA, Carcinoma; CHD, Coronary Heart Disease; CIC, CoV2 in CSF; CM, Comorbidities; COPD, Chronic Obstructive Pulmonary Disease; DM, Diabetes, f, female; GBS*, No NCSs reported; HLP, Hyperlipidemia; IG, Immunoglobulins; LC, Lung Cancer; LOO, Latency between onset of GBS and COVID-19 respectively vice versa; m, male; nd, not done; nr, not reported; NTX, Renal Transplantation; pc, personal communication; PCB, Pharyngeal, Cervical, Brachial variant of GBS; PE, Plasma Exchange; PNC, Polyneuritis Cranialis; RA, Rheumatoid Arthritis; RI, Renal Insufficiency; RSD, Reflex Sympathetic Dystrophy, S, Steroids, & antibodies positive in CSF.

**Table 4:** Comparison of PCG patients between first and second half of 2020 and the first half of 2021.

	**1st half 2020**	**2nd half 2020**	**1st half 2021**	**Total**
**Number of publications**	25	74	25	124
**Number of patients (n)**	33	192	75	300
**Age (years)**	21‒84 (28/33)	8‒94 (192/192)	7‒81 (75/75)	7‒94
**Sex**				
M	18	133	50	201
F	10	57	25	92
Nr	5	2	0	7
**A/B**				
After	30	131	72	233
Before	2	4	1	7
Together with	1	2	0	3
Nr	0	55	2	57
**Latency (days)**	−9 to 24	−10 to 90	1‒42	−10 to 90
**Subtypes**				
AIDP	22	94	55	171
AMAN	4	11	9	24
AMSAN	2	9	5	16
MFS	2	6	0	8
PNC/MNC	0	3	0	3
PCB	0	1	0	1
Nr	3	68	6	77
**CSF SARS-CoV-2**				
Negative	16	41	11	68
Positive	0	0	1	1
Nr	17	151	63	231
**Comorbidities**^a^	6	33	11	49
**Treatment**				
IG	27	168	46	241
PE	6	14	8	28
S	1	2	4	7
None	2	6	0	8
Nr	0	5	25	30
**Mechanical ventilation**				
Yes	9	36	14	59
No	23	66	35	124
Nr	1	90	26	117
**Outcome**				
Complete recovery	9	26	7	42
Partial recovery	19	106	38	163
Death	2	10	5	17
Nr	3	50	25	78
**Comorbidity + death**	1	5	2	8
**No comorbidity + death**	1	3	1	5

MNC, Mononeuritis Cranialis; Nr, Not reported.

a Only cardiovascular, pulmonary, cerebral disease, and malignancies were encountered.

## Discussion

The review shows that PCG can be a complication of COVID-19 and suggests that SARS-CoV-2 vaccinations reduce the prevalence of PCG. Whether the prevalence of PCG has really increased since the outbreak of the pandemic is still a matter of debate. Some studies report an increase in the prevalence, others a decrease.^12^^,^^13^ There are also studies that report no change in GBS prevalence since the outbreak of the pandemic.^14^

Because CSF is devoid of viral RNA in almost all cases and because cytokines are elevated in the CSF in PCG patients,^15^ an abnormal immune response rather than an infectious cause is the most likely pathophysiology underlying the development of PCG. Because PCG recovery is incomplete at discharge in most cases, PCG has to be classified as a serious complication of COVID-19.

The surprising finding that the number of reported PCG patients was lower in the first half of 2021 compared to the second half of 2020 can be asserted by several explanations. First, scientists are no longer interested in the topic as evidence accumulates that PCG is an established complication of COVID-19. The interest in publishing established facts is therefore understandably low. Second, editors are no longer interested in publishing case reports or case series for the same reason. Third, COVID-19 patients were more severely ill than before in the first half of 2021 and therefore died prematurely before they could develop GBS. However, there is no evidence to support this speculation. In most registries, mortality from COVID-19 did not increase with the occurrence of more virulent variants of the virus. Fourth, the prevalence of PCG is actually declining either due to improved strategies to treat COVID-19 or due to the effect of vaccination. Since COVID-19 treatment has not changed and has not become more causal and effective than months before, the former speculations are rather unlikely. So if the prevalence of PCG has really decreased, a positive effect of vaccinations is conceivable.

In general, SARS-CoV-2 vaccinations not only have advantages but are sometimes accompanied by side effects, such as GBS.^16^ Whether the prevalence of GBS as a side effect of SARS-CoV-2 vaccinations is higher compared to other vaccinations or whether PSG resulted in an overall increase in GBS prevalence is a matter of controversy. In a recent population-based historical rate comparison study and self-controlled case series analysis, only 11 PSG cases were observed after the first Astra Zeneca Vaccination (AZV) dose and only 5 PSG cases after the second dose.^17^ Fewer than 5 PCG cases were reported among those who received the BioNtech Pfizer Vaccine (BPV) and no PCG cases among those who received the Johnson & Johnson Vaccine (JJV).^17^ In a recent analysis of the US Vaccine Adverse Reporting System (VAERS) fewer than 1 PCG case per 1000,000 vaccine doses were reported within 42 days of vaccination in a period from January 2021 to 14th June 2021.^18^ In this study neurological side effects were observed more frequently after use of the JJV than after the use of the BPV or the Moderna vaccine.^18^ In a recent systematic review and meta-analysis of 48 publications reporting 2110,441,600 participants, the pooled incidence of PCG was 3.09 per 1 million people within six weeks of vaccination, which corresponds to 2.47 cases per 100,000 person year.^19^^,^^20^ The pooled incidence was higher as compared to patients who received the influenza vaccine.^19^

Regarding the treatment of PCG, it is not at a variance of that applied in patients with non-SARS-CoV-2 associated GBS. However, it is currently unknown whether the therapy is just as effective as in non-SARS-CoV-2 associated GBS. Recently, an emerging new treatment strategy for GBS has been proposed that may affect the prevalence of PCG (“zipper strategy”).^17^ The approach is based on the combination of IVIG alternating with PE.^17^ The therapy is based on the idea that PE eliminates the autoantibodies and cytokines and administering IVIG immediately after PE neutralizes those antibodies that are newly formed or transited from tissue. The subsequent PE session eliminates the antibodies away.^17^ This new approach can improve the outcome of PCG patients. Since PCG strongly influences the outcome of SARS-CoV-2 infections and since the outcome of PCG is worse among those with than without comorbidities, PCG needs to be recognized early and comorbidities sufficiently treated to improve the overall outcome of COVID-19 patients.

### Limitations

A limitation of the study is that publication dates do not necessarily reflect the dates when patients were diagnosed and treated. A further limitation is the design. A prospective, multicentre design is more appropriate than a retrospective design to assess a putative vaccination effect. Among the 7 patients in whom GBS seemingly preceded the viral infection, symptoms of the infection were either not adequately acknowledged or the infection initially remained asymptomatic.

### Future directions

Future studies should focus on the question if SARS-CoV-2 vaccinations really reduce the frequency of SARS-CoV-2 infection-associated complications. More generally, they should assess if vaccinations improve the outcome of COVID-19 and reduce the rate of long-COVID, the frequency, and duration of hospitalizations, including the Intensive Care Unit (ICU), and if they reduce mortality.

## Conclusions

The present study enriches the current literature as it shows that the prevalence of PCG appears to have decreased since the introduction of SARS-CoV-2 vaccines. To assess if SARS-CoV-2 vaccinations really reduce the prevalence of PCG, prospective, multicentre studies are urgently needed. If such studies confirm the results of the index study, vaccinations should be advocated and encouraged provided they are safe for everyone.

## Declarations

Ethics approval and consent to participate: not applicable

Consent for publication: not applicable

Availability of data and material: all data reported are available from the corresponding author

Funding: none received

## Conflicts of interest

The authors declare no conflicts of interest.
